# Effects of Soaking and Fermentation Time on Biogenic Amines Content of *Maesil* (*Prunus Mume*) Extract

**DOI:** 10.3390/foods8110592

**Published:** 2019-11-19

**Authors:** So Hee Yoon, Eunmi Koh, Bogyoung Choi, BoKyung Moon

**Affiliations:** 1 Department of Food and Nutrition, Chung-Ang University, Gyeonggi-do 17546, Korea; kisingserpe@naver.com; 2 Major of Food & Nutrition, Division of Applied Food System, Seoul Women’s University, Seoul 01797, Korea; kohem7@swu.ac.kr (E.K.); 5326460@hanmail.net (B.C.)

**Keywords:** biogenic amine, *maesil*, amino acids, soaking, fermentation, temperature

## Abstract

*Maesil* extract, a fruit-juice concentrate derived from *Prunus mume* prepared by fermenting with sugar, is widely used with increasing popularity in Korea. Biogenic amines in *maesil* extract were extracted with 0.4 M perchloric acid, derivatized with dansyl chloride, and detected using high-performance liquid chromatography. Among 18 home-made *maesil* extracts collected from different regions, total biogenic amine content varied from 2.53 to 241.73 mg/L. To elucidate the effects of soaking and fermentation time on biogenic amine content in *maesil* extract, *maesil* was soaked in brown sugar for 90 days and the liquid obtained was further fermented for 180 days at 15 and 25 °C, respectively. The main biogenic amines extracted were putrescine and spermidine and the total biogenic amine content was higher at 25 °C than at 15 °C. Soaking at 15 and 25 °C increased the total biogenic amines content from 14.14 to 34.98 mg/L and 37.33 to 69.05 mg/L, respectively, whereas a 180 day fermentation decreased the content from 31.66 to 13.59 mg/L and 116.82 to 57.05 mg/L, respectively. Biogenic amine content was correlated with total amino acid content (particularly, arginine content). Based on these results, we have considered that biogenic amine synthesis can be reduced during *maesil* extract production by controlling temperature and fermentation time.

## 1. Introduction

*Maesil* (*Prunus mume*) known as Japanese *Ume* has been used not only as a food but also as a medicine on account of its various functionalities [1,2,3]. As the seed of *maesil* has a toxic substance called amygdalin [4], *maesil* has been processed into various products such as alcoholic beverage, juice, pickle or extract rather than eaten raw [5]. *Maesil* extract is a fruit-juice concentrate produced by the fermentation of *maesil* and sugar. Recently, it has been increasingly used as a seasoning to impart sweetness and a unique flavor to foods [5,6,7,8]. Traditionally, *maesil* extract is soaked for a long period (90 days) at room temperature and fermented naturally under different conditions in individual households. Therefore, uncontrolled fermentation can lead to the formation of biogenic amines, which are produced by molds and bacteria.

As biogenic amines are mainly produced by the microbial decarboxylation of free amino acids, they are easily found in fermented foods [9,10]. These biogenic amines have been reported to be abundant and they have been found in a wide range of food products, including fish products, soy sauce, *Chunjang* (traditional fermented soybean paste in Korea and China), and agricultural products [11,12,13,14,15]. As a high intake of biogenic amines can cause various detrimental effects such as migraine and gastrointestinal problems, their ingestion needs to be restricted [16,17]. Indeed, their content is currently regulated in certain food products. For example, the histamine content in fish products is regulated by the US Food and Drug Administration (FDA, 50 mg/kg) and the European Union (100 mg/kg) in fish products [18]. The formation of biogenic amines is influenced by microbial flora and their growth as well as the fermentation conditions used in the production of fermented foods [19,20]. To date, however, studies on the changes in biogenic amines during fruit fermentation have mainly focused on wine [9]. Moreover, little research has been conducted on the fermentation of biogenic amines during the fermentation of other fruits.

Therefore, in this study, we tried to monitor the biogenic amine content of *maesil* extracts and determine the effect of fermentation conditions on the changes in biogenic amines in *maesil* extracts during fermentation. For this purpose, we (i) determined the content of biogenic amines content in 18 home-made *maesil* extracts collected from different households in Korea and (ii) monitored the content of biogenic amines during the fermentation of *maesil* extracts at two different temperatures, 15 and 25 °C, over a period of 9 months. 

## 2. Materials and Methods 

### 2.1. Chemicals

Biogenic amine standards (histamine dihydrochloride (HIS), tryptamine hydrochloride (TRP), 2-phenylethylamine (2-PHE), putrescine dihydrochloride (PUT), cadaverine dihydrochloride (CAD), tyramine hydrochloride (TYR), spermidine trihydrochloride (SPD), and spermine tetrahydrochloride (SPM)) and dansyl chloride were obtained from Sigma-Aldrich Chemical Co. (St. Louis, MO, USA). Perchloric acid, 25% ammonium hydroxide solution, sodium hydroxide sodium hydrogen carbonate, and diethyl ether were acquired from Daejung Chemical Co. (Siheung, Korea). Acetone and acetonitrile (High-performance liquid chromatography (HPLC) grade) were purchased from Tedia Co. (Fairfield, OH, USA). Compound mixtures of amino acids, borate buffer, *o*-phthalaldehyde (OPA) and 9-fluorenylmethoxycarbonyl chloride (FMOC-Cl) were obtained from Agilent Technologies (Andover, MA, USA). 

### 2.2. Preparation of Food Samples 

**Figure 1:**
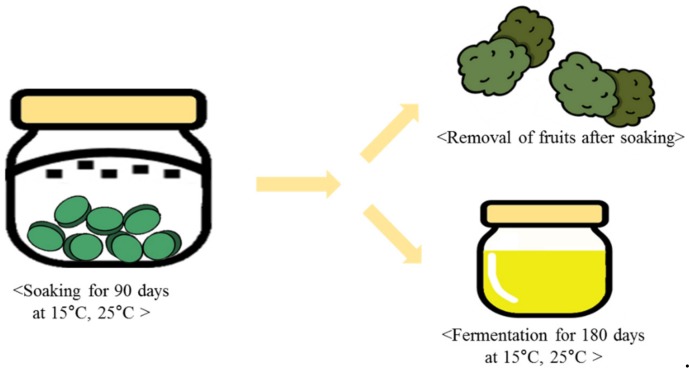
The fermentation process used for producing *maesil* extract.888.

During the period from 2010 to 2014, we collected samples 18 *maesil* extracts from different households in Korea for analysis of biogenic amines content. We also prepared our own *maesil* extract, following the method of Choi and Koh (2016) [5], and the process of preparation is shown in Figure 1. *Maesil* fruits obtained from a local market were washed with pure water, and drained at room temperature (23 ± 1 °C). To the 400 g of *maesil*, we added 400 g of brown sugar and the mixture was then placed in 1 L clear plastic jars, which were maintained in incubators set at 15 °C and 25 °C, respectively. After the *maesil* fruits were taken out from the jar after 90 days of soaking, the obtained liquid (490 mL at 15 °C and 476 mL at 25 °C) was further fermented for the next 180 days in the same jar. Biogenic amines were analyzed at 30, 45, 75, and 90 days of soaking period and 30, 60, 120, 150, and 180 days of fermentation. 

### 2.3. pH Measurement 

To measure the pH, *maesil* extract (10 g) was mixed with 10 mL deionized water for 3 min and then filtered through Whatman paper No.2 filter paper (Advantec, Tokyo, Japan). The pH was measured using a pH meter (Beckman Coulter, FL, USA) following the method of Shukla et al. [21]. 

### 2.4. Amino Acids Analysis 

Amino acids in the *maesil* extract were analyzed using an HPLC system (Dionex Ultimate 3000, Thermo Fisher Scientific, Waltham, MA, USA), equipped with a 1260 Infinity fluorescence detector (Agilent Technologies, Waldbronn, Germany), following the method described by Jajic et al. (2013) [22] with slight modifications. The samples were derivatized with OPA and FMOC via a programmed autosampler. After derivatization, samples (0.5 µL) were injected into an Inno-C_18_ column (4.6 × 50 mm, 5 µm, Youngin Biochrom, Korea) at 40 °C. The fluorescence was detected at excitation and emission wavelengths of 340 and 450 mm, respectively for OPA, and at 266 and 305 nm, respectively, for FMOC. Primary and secondary amino acids were analyzed based on the OPA and FMOC derivatives, respectively. The mobile phase solvent A was 40 mM sodium phosphate (pH 7), and solvent B was a 10:45:45 (*v/v*) mixture of distilled water, acetonitrile, and methanol. The gradient program was run at a flow rate of 1.0 mL/min as follows: 5% B for 3 min; followed by elution with 5% to 55% B in 24 min; 55% to 90% B in 25 min; maintained 90% of B for next 6 min; and 90% to 5% B for 3.5 min, maintained for 0.5 min. 

### 2.5. Biogenic Amine Analysis

#### 2.5.1. Extraction of Biogenic Amines 

Biogenic amines were extracted from the *maesil* extract using the method of Shukla et al. (2014) [21] with slight modifications. Briefly, 10 mL of 0.4 M perchloric acid solution was mixed with 5 g *maesil* extract, homogenized for 3 min, and then centrifuged at 3000× *g* for 10 min at 4 °C. The residue was re-extracted with 0.4 M perchloric acid solution (10 mL). After the supernatants were combined and 0.4 M perchloric acid solution was added to adjust the final volume to 50 mL. After filtering through Whatman filter paper No.1 (11 µm, Adventec, Tokyo, Japan), 1 mL of the extract was used for derivatization with dansyl chloride.

#### 2.5.2. Derivatization of Biogenic Amines 

Biogenic amines were derivatized following the methods described by Shukla et al. (2010) [23] and Frias et al. (2007) [24]. An extract sample (1 mL) or standard solution mixture (1 mL) was mixed with 200 μL 2 M sodium hydroxide; next, 300 µL of sodium hydrogen carbonate solution was added to saturate the solution. To the mixture, 1 mL of a dansyl chloride solution (10 mg/mL in acetone) was added and kept for 45 min at 40 °C. To stop the reaction, 100 µL of 25% ammonium hydroxide was added to the mixture and reacted for 30 min at 25 °C. Then, the derivatized biogenic amines were extracted twice with 1 mL of diethyl ether. Subsequent to drying in a nitrogen stream, the extract was redissolved in acetonitrile (1 mL) and filtered through a 0.22 µm polyvinylidene fluoride (PVDF) filter (Millipore Co., Bedford, MA, USA) for injection into the HPLC system.

#### 2.5.3. HPLC Analysis of Biogenic Amines 

Biogenic amines were analyzed using an HPLC system consisting of an Alliance 2695 separations module (Waters, Milford, MA, USA) and Ultra violet (UV)/Visible detector 2487 (Waters, Milford, MA, USA) with a Capcell Pak C18 column (4.6 × 250 mm i.d., 5 µm; Shiseido, Kyoto, Japan), thermostated at 30 °C, and detected at 210 nm [24,25,26]. The injection volume was 20 µL and the mobile phase consisted of solvent A (water) and B (acetonitrile) run at a flow rate of 0.8 mL/min with the following gradient elution program for 35 min: 65:35 (A:B, *v*/*v*), followed by 45% B for 5 min, elution with 45% to 65% B in 10.05 min, 65% to 80% B in 17.05 min, 80% to 90% B up to 26.25 min, and 90% to 35% B in 35 min.

### 2.6. Method Validation 

The HPLC method for biogenic amines analysis was validated for linearity, limits of detection (LOD) and limits of quantification (LOQ), accuracy, and precision [22]. The linearity was evaluated using five concentrations (0.5, 1, 2, 5, and 10 mg/L) of each the biogenic amine standards (PUT, CAD, HIS, TRP, 2-PHE, TYR, SPD, and SPM) by constructing a calibration curve. The LOD and LOQ values were calculated using the following equations: LOD = 3.3 × (standard deviation (SD)/slope of calibration curve) and LOQ = 10 × (SD/slope of calibration curve). The accuracy of the method was verified by triplicate analysis of spiked samples at two different levels (5 and 10 mg/L) and expressed as % recovery. The recoveries were calculated by contrasting the peak area of measured concentration with the peak area of the spiked concentrations. To evaluate the precision, repeatability, inter-day, and intra-day were performed and expressed as the percentage relative standard deviation (RSD) of the peak area measurements. Repeatability was estimated by analysis of six consecutively injected samples. The inter-day precision was determined at two different levels, 5 and 10 mg/L, and the analyses were performed over a period of three consecutive days. The intra-day precision was determined by spiking five blank samples at concentrations levels of 5 and 10 mg/mL and the evaluation was based on the results obtained using the method operating over a single day under the same conditions. 

### 2.7. Statistical Analysis 

Quantitative data are expressed as the means ± SD of at least three measurements. Statistical analysis was performed using a one-way analysis of variance (ANOVA) and Duncan’s multiple range test by SAS software, version 8.0 for Windows (SAS Institute, Cary, NC, USA). The probability value of *p* < 0.05 was considered statistically significant.

## 3. Results and Discussion

### 3.1. Method Validation

The results obtained from the different method validations are presented in Table 1. Standard curves for biogenic amines were constructed from triplicate analyses of five concentrations in the range 0.5–10 mg/L. With the exception of spermine (correlation coefficient (R^2^) > 0.998), the linearity of the calibration curves for each biogenic amine was >0.999. The precision, expressed as %RSD, of inter-day variation was between 0.17% and 5.20%, and the RSD values for intra-day variation were between 0.07% and 6.46%. The LOD and LOQ of the biogenic amines ranged from 0.01 to 0.20 mg/L and 0.02 to 0.61 mg/L, respectively. The accuracy of the method with regard to recovery was between 89.4% and 110.8%. 

### 3.2. Content of Biogenic Amines in Home-Made Maesil Extract 

Among the 18 home-made maesil extracts analyzed, the total content of biogenic amines ranged from 2.5 to 241.7 mg/L, the major individual biogenic amines were putrescine (not detectable (ND)-80.82 mg/L) and spermidine (ND-219.20 mg/L), followed by tryptamine (Table 2). Putrescine, histamine, tyramine, cadaverine, 2-phenylethylamine, spermidine, spermine, agmatine, and tryptamine are the main biogenic amines in wine [27]. Among these amines, putrescine has been reported to be generated from the raw material or by microbial decarboxylation [28]. In the case of wine, putrescine content has been found to be influenced by geographical region and grape variety [29]. Histamine and spermine detected in wine [27,29,30] are known to have toxicity or play a role in enhancing toxicity [11,31]. However, we were unable to detect either of these two amines in the 18 maesil extracts examined in the present study. These results imply that the amount and composition of biogenic amines may differ widely among different fruit-derived products and that these differences could be attributed to differences in manufacturing practice and fruit material.

The content of biogenic amines is known to be affected by fermentation conditions, including temperature, microorganisms, and the synthetic pathways of the biogenic amine formation [32,33,34,35]. In wine, cadaverine, histamine, putrescine, and tyramine are mainly detected, the content of which can vary depending on fermentation factors, storage, microbial decarboxylase activity, and vinification [27,30,36]. Marcobal et al. (2006) have reported that the content of biogenic amines in wine ranged from ND to 54.02 mg/L [31]. Garai et al. (2006) found that the main biogenic amine in commercial apple ciders was putrescine and that the total biogenic amine content ranged from ND to 23.26 mg/L [12]. In comparison, the results of this study indicate that the biogenic amine content in home-made *maesil* extracts is considerably higher than that reported in wine or apple ciders [12,27,30], thereby emphasizing the necessity to control biogenic amines productions during the fermentation of *maesil* extract. 

### 3.3. Content of Biogenic Amines During Soaking and Fermentation

**Figure 2:**
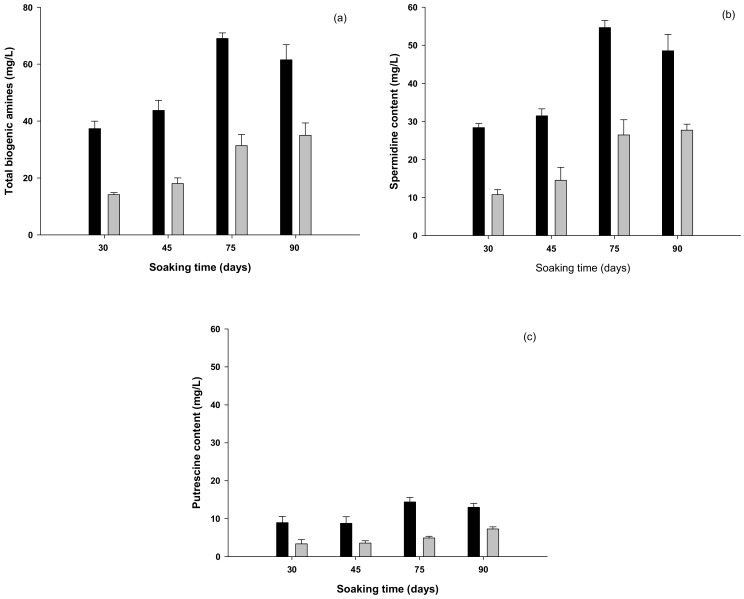
The content (mg/L) of total biogenic amines (**a**), spermidine (**b**), and putrescine (**c**) during soaking at 25 °C (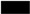) and 15 °C (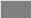).

During the 90 day soaking of *maesil* examined in the present study, we found that the total biogenic amines content increased from 14.1 to 35.0 mg/L and 37.3 to 69.1 mg/L at 15 and 25 °C, respectively, indicating that the content was higher at the latter temperature throughout the soaking period (Figure 1a). Previous studies have reported that biogenic amines are generated via the catalytic activity of decarboxylase enzymes produced during the growth of microorganisms such as lactic acid bacteria [37], and thus, the increase in biogenic amines during the soaking period might be caused by microbial decarboxylase activity. At both incubation temperatures we assessed, the predominant biogenic amines detected in *maesil* extract were putrescine and spermidine (Figure 2b,c), and the latter comprised approximately 80% of the total biogenic amines. 

After removing the *maesil* fruit from the sample jars at the end of the soaking period, the residual liquid was subsequently fermented for 180 days, during which, the content of biogenic amines decreased from 31.7 to 13.6 mg/L and 116.8 to 57.1 mg/L at 15 and 25 °C, respectively (Table 3). Generally, the extracts fermented at 25 °C exhibited biogenic amines content that was twice as high as that obtained at 15 °C. Moreover, at the end of the fermentation period, the total biogenic amines content at 15 °C was 23.8% of that at 25 °C. In addition to putrescine and spermidine, tryptamine was also detected at 0.33 mg/L when *maesil* was fermented at the higher temperature for 30 days. In this regard, Chong et al. (2011) have reported that temperature was the most important factor affecting biogenic amines formation [38], and Pinho et al. (2001) reported a higher increase in biogenic amines at a storage temperature of 21 °C than at 4 °C [35]. In addition, Kim et al. (2002) found that 25 °C was the optimum temperature for histamine production in fish muscles [39]. *Maesil* extract is typically produced by natural fermentation without controlling the temperature or starter culture. Moreover, it is sometimes consumed immediately after a 90 day soaking without subsequent fermentation. On the basis of the results obtained in this study, we recommend that, to yield a product with lower levels of biogenic amines, *maesil* extract should be fermented at a relatively low temperature and for a long period of time. 

### 3.4. Effects of Processing Factors on Biogenic Amines Formation 

The pathways implicated in the synthesis of biogenic amines can vary depending on the temperature, sugar content, precursors, and microorganisms involved in the fermentation of various food items [16,32,40]. Generally, putrescine is derived from the decarboxylation of arginine and ornithine or is already present in raw materials [28,32], whereas, spermidine is produced from arginine and ornithine or is converted from putrescine by spermidine synthase [16,31]. Poveda (2019) and Bardocz (1995) reported that most putrescine is either converted to spermidine or spermine, or is catabolized to succinate and other amino acids via succinate [28,31].

In the present study, to determine the effects of the factors influencing biogenic amines formation, we performed Pearson’s correlation. Among these factors, we detected no significant correlation between biogenic amines content and pH, which had a narrow range (pH 2.9–3.3) during the fermentation. 

**Figure 3:**
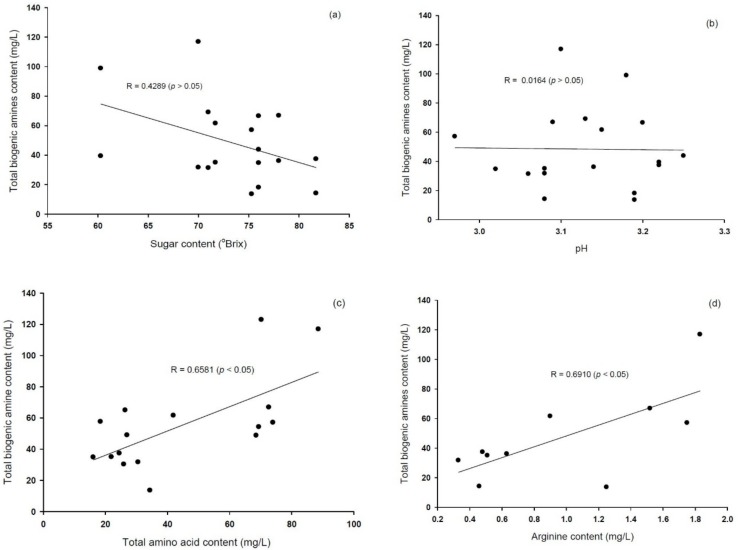
Correlations between sugar content and biogenic amine content (mg/L) (**a**); pH and biogenic amine content (mg/L) (**b**); the content (mg/L) of total amino acid and total biogenic amines (**c**); and the content (mg/L) of biogenic amines and arginine (**d**).

Arena et al. (2008) reported a negative correlation between biogenic amines and sugar concentration and found that; the additions of glucose and fructose at 5 and 20 g/L reduced biogenic amines production by 82%–93% and 61%–99%, respectively [40]. Cid et al. (2008) reported that lower glucose concentration is associated with a high activity of ornithine-decarboxylase produced by *Lactobacillus* [41]. In our study, we found that, although there was a negative correlation between sugar content and biogenic amines content (Figure 3a), the relationship was not significant, which could be attributable to the narrow range of sugar content (61 to 81 °Brix) during fermentation.

The total biogenic amines content showed a positive correlation with the total amounts of amino acids (R = 0.6581, *p* < 0.05), which could be explained by the fact that amino acids are precursors of biogenic amines [42]. We also detected a strong positive correlation between the amounts of putrescine and spermidine (R = 0.9277, *p* < 0.01; data not shown), consistent with the findings of Bardocz (1995) [31] and Nuriez et al. (2016) [16], which indicates that putrescine is a precursor of spermidine. However, apart from a positive correlation between arginine and total biogenic amines content (R = 0.6910, *p* < 0.05), we detected no correlation between individual biogenic amines and their respective precursor amino acid, which is consistent with the findings reported by Soufleros et al. (1998) [43]. Gezginc et al. (2013) reported that arginine serves as a precursor of putrescine, which can, in turn, be converted to spermidine [32]. Furthermore, it has been found that, in plants and some microorganisms, there are alternative pathways in which putrescine is generated from arginine via agmatine [33]. These results indicate that the fermentation of *maesil* extract at low temperature could reduce the production of biogenic amines. In addition, biogenic amine formation in *maesil* extract could be affected by the origin of *maesil*, the number of amino acids as well as the content of biogenic amine precursors. 

## 4. Conclusions

The present study was conducted to evaluate the changes in biogenic amines formation and the relationship between biogenic amines and amino acids in *maesil* extract during the fermentation of this product. Although the consumption of *maesil* extract is currently increasing, there has, to date, been a lack of studies on the changes that biogenic amines undergo during *maesil* extract fermentation. The results of this study showed that the biogenic amines content in *maesil* extract is affected by both the inherent amino acids content and fermentation temperature and time. Moreover, the content of some biogenic amines may also be affected by the presence of other biogenic amines. We found that both amino acids and biogenic amines content was lower during fermentation at 15 °C than at 25 °C and decreased with increasing fermentation time. Accordingly, these observations indicate that employing protracted low-temperature fermentations could be an effective approach for reducing the production of biogenic amines in *maesil* extract. In further research, it will be necessary to study the types of microorganisms and formation on biogenic amines in *maesil* extract.

## Figures and Tables

**Table 1:** Summary results relating to validation of the high-performance liquid chromatography (HPLC) method used for biogenic amines.

Compound	R^2^	LOD (mg/L)	LOQ (mg/L)	Precision (%RSD)				Accuracy	
				Inter-Day		Intra-Day		Recovery (%)	
				Low	High	Low	High	Low	High
HIS	1.0000	0.02	0.04	3.82	5.20	0.49	6.46	97.9	89.4
TRP	1.0000	0.01	0.02	0.66	0.19	0.10	0.09	101.8	105.3
2-PHE	1.0000	0.16	0.22	0.22	0.74	0.07	0.64	101.3	97.9
PUT	1.0000	0.01	0.02	0.45	0.34	0.25	0.76	99.8	104.0
CAD	1.0000	0.01	0.02	0.14	0.19	0.25	0.49	100.1	100.0
TYR	1.0000	0.07	0.10	0.27	0.17	0.09	0.22	99.7	99.3
SPM	0.9981	0.20	0.61	2.27	1.86	1.07	2.21	95.1	110.8
SPD	1.0000	0.04	0.08	0.14	0.40	0.16	0.11	107.1	99.6

LOD: Limits of detection; LOQ: Limits of quantification; RSD: Relative standard deviation HIS: Histamine; TRP: Tryptamine; PUT: Putrescine; 2-PHE: 2-phenylethylamine; CAD: Cadaverine; TYR: Tyramine; SPM: Spermine; SPD: Spermidine.

**Table 2:** Biogenic amines content (mg/L) in 18 home-made *maesil* extracts prepared in individual households.

Sample	HIS	TRP	2-PHE	PUT	CAD	TYR	SPM	SPD	Total
A	ND	ND	ND	19.7 ± 0.53	2.9 ± 1.01	ND	ND	219.2 ± 6.30	241.8 ± 4.89
B	ND	3.0 ± 0.53	ND	12.4 ± 0.67	ND	ND	ND	44.5 ± 1.96	60.0 ± 1.64
C	ND	ND	ND	15.3 ± 0.74	ND	ND	ND	14.9 ± 0.91	30.2 ± 1.64
D	ND	1.9 ± 0.36	ND	8.8 ± 0.42	ND	ND	ND	25.6 ± 0.74	36.2 ± 0.18
E	ND	3.2 ± 0.47	ND	15.9 ± 1.69	ND	ND	ND	44.3 ± 2.31	63.4 ± 4.07
F	ND	ND	ND	13.1 ± 0.99	ND	ND	ND	83.5 ± 6.28	96.5 ± 6.21
G	ND	2.9 ± 0.65	ND	25.8 ± 2.33	17.3 ± 3.79	ND	ND	44.6 ± 3.11	90.6 ± 4.93
H	ND	5.7 ± 1.32	ND	ND	12.7 ± 0.42	ND	ND	35.9 ± 1.22	54.3 ± 2.09
I	ND	2.5 ± 0.48	ND	ND	ND	ND	ND	ND	2.5 ± 0.48
J	ND	3.5 ± 0.35	5.26 ± 1.47	ND	ND	ND	ND	ND	8.8 ± 1.19
K	ND	5.3 ± 0.45	ND	21.8 ± 0.70	ND	0.8 ± 0.02	ND	77.9 ± 1.81	105.8 ± 1.38
L	ND	ND	ND	ND	ND	1.0 ± 0.12	ND	ND	1.0 ± 0.12
M	ND	5.4 ± 0.38	ND	ND	ND	ND	ND	ND	5.4 ± 0.38
N	ND	ND	ND	17.5 ± 0.15	ND	ND	ND	71.8 ± 4.60	89.3 ± 4.45
O	ND	3.2 ± 035	ND	6.8 ± 0.26	ND	ND	ND	ND	9.9 ± 0.46
P	ND	ND	ND	80.8 ± 4.72	ND	ND	ND	17.1 ± 1.76	97.9 ± 4.29
Q	ND	4.1 ± 0.83	ND	5.9 ± 0.70	ND	0.7 ± 0.07	ND	10.1 ± 1.44	20.8 ± 3.10
R	ND	3.4 ± 0.45	ND	8.4 ± 0.97	ND	ND	ND	41.3 ± 1.41	53.1 ± 2.01

HIS: Histamine; TRP: Tryptamine; PUT: Putrescine; 2-PHE: 2-phenylethylamine; CAD: Cadaverine; TYR: Tyramine; SPM: Spermine; SPD: Spermidine; ND: Not detected.

**Table 3:** Biogenic amines content (mg/L) during fermentation at different temperatures.

	Fermentation Days at 15 °C					Fermentation Days at 25 °C				
Biogenic amine	30	60	120	150	180	30	60	120	150	180
TRP	ND	ND	ND	ND	ND	0.33 ± 0.09	ND	ND	ND	ND
PUT	8.1 ± 1.18 ^a^	12.8 ± 1.15 ^b^	13.8 ± 0.67 ^b^	12.5 ± 1.95 ^b^	7.4 ± 0.01 ^a^	30.9 ± 0.77 ^c^	23.9 ± 1.56 ^b^	14.5 ± 0.76 ^a^	20.0 ± 0.34 ^b^	15.6 ± 4.03 ^a^
CAD	ND	ND	ND	ND	ND	ND	ND	ND	ND	ND
SPD	23.6 ± 1.19 ^b^	26.6 ± 2.32 ^c^	22.2 ± 0.66 ^b^	22.2 ± 0.65 ^b^	6.2 ± 0.74 ^a^	84.8 ± 0.68 ^d^	74.9 ± 2.36 ^c^	52.3 ± 3.25 ^b^	46.5 ± 4.93 ^a,b^	41.4 ± 2.58 ^a^
Total	31.7 ± 2.15 ^b^	39.4 ± 3.30 ^d^	36.0 ± 1.33 ^c,d^	34.7 ± 2.69 ^b,c^	13.6 ± 0.75 ^a^	116.8 ± 4.17 ^d^	98.8 ± 0.93 ^c^	66.8 ± 3.96 ^b^	66.5 ± 4.59 ^b^	57.0 ± 6.43 ^a^

TRP: Tryptamine; PUT: Putrescine; CAD: Cadaverine; SPD: Spermidine; ND: Not detected. Each value expressed the mean of triplicates ± standard deviation (SD). Different superscript letters in the same row indicate significant difference (*p* < 0.05).
